# On the Pathology of the Articulating Cartilages

**Published:** 1839-04

**Authors:** 


Art. X.—*De Cartilaginum Articularium ex Morbis Mutationes.* Auct. L. H. Schumer, Jr.—*Groningæ*, 1836. 8vo. pp. 64.

*On the Pathology of the Articulating Cartilages.* By L. H. Schumer, Jun. m.d.—*Groningen*, 1836. 8vo. pp. 64.

The main question discussed in this essay is, whether the cartilages possess in themselves sufficient vitality for carrying on the processes of inflammation, ulceration, &c., or whether the changes, that observation shows to occur in them, are simply induced by the material action of disease and its consequences affecting the adjoining tissues. The author commences by an enumeration of the doctrines of a host of writers, which, according to him, afford a melancholy specimen of that triumph of theory over fact, of speculation over the evidence of the senses, so often encountered in tracing the progress of medical knowledge: for, he asserts that, so long since as 1798, Dörner distinctly proved the almost total impossibility of inflammation affecting the articular cartilages; yet inflammation of those bodies has since then been regularly admitted into nosological systems, and its effects dwelt on by a variety of writers. We cannot here enter into an examination of this thorny debate, but will give the reader a brief abstract of the results which induce Dr. Schumer to deny to the cartilages themselves all active participation in the changes they undergo. 1. The knee-joint of a goat was cut open transversely, and the femoral cartilages and synovial membrane touched in several places with black ink. The black colour instantly penetrated into the cartilage, and it was found impossible to wipe it away. The animal was killed eight days after, and the blackness of the cartilage found unchanged: none was discernible in the synovial membrane. Precisely the same results followed when the experiment was made on a piece of *dead* cartilage. 2. The tibia of a sheep was removed from its femoral articulation, and the wound dressed in the ordinary manner. The animal was killed on the ninth day after the operation, and the femoral cartilage was found unchanged, although the synovial membrane was inflamed and covered with false membrane. This experiment was varied in other animals, by chopping and cutting away pieces of the cartilage, and exposing its surface constantly to the air; but invariably with the same result: neither in colour nor in vascularity did the least change appear in its condition. By injecting the femoral artery with mercury, a multitude of vessels were shown in the circumjacent soft parts and in the extremity of the *bone;* but not a single globule of the metal entered the cartilage itself, not even the surface in connexion with the femur. The conclusion deducible from these experiments is, according to the author, very plain.

But if not produced by inflammation of their own substance, how are the changes observed in those tissues effected? That the opacity and yellowish tinge in the cartilages of inflamed joints is produced simply by the absorption (imbibition) of pus appears from an experiment by Professor Sebastian. He introduced a fragment of cartilage into a fistulous canal filled with pus: in seven days it had grown opaque, yellowish, and had lost half its bulk. The next change ascribed by writers to inflammation is that usually styled *ulceration* of the cartilages. The destruction of tissue in these cases proceeds either from the free surface of the cartilage towards the bone, or from the latter towards the former. In the first instance the cavity is filled with fluid, usually pus, sometimes perhaps diseased synovia. But as, by the experiment above related, it is shown that dead cartilage may be dissolved in the short space of seven days, there can be little doubt but that erosions of the living substance are similarly produced, inasmuch as the living and dead tissues in nowise differ from each other as respects the imbibition of fluids. An analogous process of chemical solution sometimes destroys the epidermis, and is also the cause of the disappearance of the crystalline lens, when pushed into the anterior chamber. Of the second species little is said: the pus or other solvent fluid *must*, according to the writer, in these cases (which are doubtless very rare), exist between the bone and cartilage; but he does not state that he has actually found it so situated.

Softening of the cartilages, or chondromalacia, is also attributed to imbibition of pus. Some intelligent remarks on anchylosis follow; and the whole essay, though some of the experiments detailed may admit of a different explanation from that assigned them, is creditable to the research and acumen of its author.

